# Fabrication and characterization of eco-friendly composite materials from natural animal fibers

**DOI:** 10.1016/j.heliyon.2021.e06954

**Published:** 2021-05-05

**Authors:** Md. Farhad Ali, Md. Sahadat Hossain, Samina Ahmed, A.M. Sarwaruddin Chowdhury

**Affiliations:** a Institute of Leather Engineering and Technology, University of Dhaka, Dhaka, 1000, Bangladesh; b Glass Research Division, Institute of Glass & Ceramic Research and Testing, Bangladesh Council of Scientific and Industrial Research (BCSIR), Dhaka, 1205, Bangladesh; c BCSIR Laboratories Dhaka, Bangladesh Council of Scientific and Industrial Research (BCSIR), Dhaka, 1205, Bangladesh; d Applied Chemistry and Chemical Engineering, Faculty of Engineering and Technology, University of Dhaka, Dhaka, 1000, Bangladesh

**Keywords:** Cow hair fiber, Environment, Composite, Mechanical properties, Resin

## Abstract

A large amount of useless portion, which is discharging from leather industries, pollutes the environment immensely. The discarded part needs to be recycled to yield other valuable products to subside environmental pollution. In this research, we utilized a rejected part (cow hair) of leather industries and unsaturated polyester resin (UPR) to fabricate valued composites to confine atmospheric pollution. A hand-lay-up technique, which is an easy and economical approach, was employed for composites fabrication. A number of cow hair fiber percentages (2, 5, 7, 10, 12, 15, and 20% by weight) were taken into consideration to investigate the impact of fiber loading on composites. Prepared composites were characterized by a few mechanical properties such as tensile strength (TS), tensile modulus (TM), elongation at break (EB), bending strength (BS), and bending modulus (BM). Fourier Transform Infrared (FTIR), Electron Scanning Microscopy (SEM), and water absorption were also performed to support the data in favor of mechanical properties. Optimum mechanical properties which were supported by the other analysis were achieved for 5% cow hair containing composites.

## 1 Introduction

Based on the sources, natural fibers are classified into plant, animal, and mineral fibers. Plant-based fibers are mainly composed of cellulose on the other hand animal-based fibers are composed of protein. Contrary, the mineral fibers are inorganic metals-based fibers [1, 2, 3, 4]. Several authors have shown that the hair has very strong physical and mechanical properties. This peculiar hair activity is due to the presence of structural proteins keratin in hair fibers [5, 6]. Hair is a biomaterial filament made mostly of keratin proteins [7, 8, 9]. These keratins are used in the mid-filament network of epithelial cell cytoplasms and can assist cells and tissues grow, shape cells, create flesh, heal wounds, and reformulate the tissues [8]. The mechanical property of the hair fiber is strongly determined by the feature of the keratin system [8, 10, 11].

Unsaturated polyesters are widely used for the structural sections of the ships such as hulls, pipes, and compartments, and are normally reinforced by fiber-glass or ground minerals. Unsaturated polyesters are polyesters consisting of saturated dicarboxylic acid or anhydride (generally phthalic anhydride), or an unsaturated dicarboxylic acid and anhydride (usually maleic anhydride). Both of these acid constituents are reacted with one or more alcohols such as ethylene glycol and propylene glycol to form long chain-like polyester molecules [12]. Notwithstanding the competition from advanced polymers, UPR remains a significant plastic degree. Applications of UPR are commonly used in plastic, wood paint, fillers, stuccos, masts and chemical anchoring, self-extinguishing mixed material, granite, marble and artificial stone, wood coats, ribbed walls, ribbed walls, acrylic coatings, bathtubs, and painting bags [13]. Many methods are available in current literature to eventually solve such limitations using the variety of reinforcements from microfibers to nanotubes in the UPR matrix [14, 15, 16, 17].

The latest research has demonstrated that the waste natural fiber can be processed to generate biodegradable, cheap, available, sustainable, and durable polymer composites without compromising the fundamental properties of fibers [18, 19]. Scientists mainly from developed countries have recently re-evaluated the economic importance of hair fiber to fabricate polymer composite substances and have been able to pioneer in a new field of industrial applications of hair fibers [20, 21].

The composites shaped by goat and horse-hair fibers in the BC age have been used to stabilize mud and mortar [22, 23, 24]. The development of synthetic and mineral fiber in the intervening decades, however, led to the intermittent practice of hair fiber as reinforcement [25]. These latest fibers have shown tremendous advances and have flooded the fiber industry in a short time, but their shortcomings in terms of production, processing, pecuniary feasibility, and environmental effect eventually obligated engines and material scientists from this modern era to rethink the prospective consumptions of animal fibers for composite [6, 26]. The key items include vehicle parts, boat hulls, engines, commercial equipment, car-body plates, tubing and baths, flooring, transparent walls, containers, resistance to corrosions, and materials of the structure. This visit to fibers, particularly keratin-based fibers, has been shown to generate a considerable number of studies with encouraging Results for composite production [27, 28]. The work carried out by Dwivedi et al. has shown that the flexural characteristics of cow hair fiber are enhanced by polyethylene and the fibers have greatly improved the mechanical features of the existing composites [6, 29]. This research aims at investigating the quality and appropriateness of the use of cow hair fibers as reinforcement in the UPR matrix. The use of cow hair fibers as a possible reinforcement leads to the ingenuity of this research.

## 2 Materials and methods

### 2.1 Materials

Unsaturated polyester resin (UPR) and methyl ethyl ketone peroxide (MEKP) were bought from the Innovative Resins Pvt. Ltd. located at Haryana-122018 in India. Hair fiber of black cow was collected from the local leather industry near hemayetpur, Savar, Dhaka, Bangladesh. The average fiber diameter (CHF) was 130 ± 15 μm and impurities, bloodstream, oil, and grade, etc., were removed from fiber by washing robustly with water and detergent. Cleaned fibers were dried at 27 °C for four days.

### 2.2 Preparation of composites

Cow hair fiber (5 mm length and 135 μm diameter) as reinforcing material and unsaturated polyester resin as the matrix were taken in a 500 ml beaker and stirred vigorously for uniform mixing. After complete mixing of reinforcing and matrix materials, a certain percentage (1%) of methyl ethyl ketone peroxide (MEKP) which was used as a hardener was added to fabricate composites. Composites were prepared by hand lay-up technique and the details of the method were reported [14]. The hair fiber percentages (by weight) were 2, 5, 7, 12, 15, and 20 where the remaining percentages were occupied by UPR. This fabrication process of the hand-lay-up technique was performed at room temperature (30 °C) in glass molds. The molds were retained for 24 h in a fume hood and then the fabricated composites are wrapped in polythene bags.

### 2.3 Mechanical properties of composites

The tensile strength (TS), tensile modulus (TM), and elongation at break (EB) were evaluated by a universal testing machine (H50 KS-0404) with an initial splitting of 20 mm, and a crosshead velocity of 10 mm/min confining sample size to 60 × 20 × 3.5 mm. The specimens were conditioned for three days before testing and all the tests were conducted under the same conditions maintaining a relative humidity of 50 percent and room temperature of 30 °C. Bending tests were also performed using the same universal testing machine to determine bending strength (BS) and bending modulus (BM). All the values documented in this manuscript are the average of at least 7 (seven) samples.

### 2.4 Water uptake

The composites were cut to a small dimension (10 × 2 × 0.2 cm) for the water uptake test. The composite samples were submerged in a beaker holding 1000 ml of distilled water at room temperature for various periods (10 min, 30 min, 2 h, 12 h, and 24 h). Samples were removed from the beaker using tissue paper. The water uptake of the composites was calculated by using the initial and final weights. The water uptake was determined by applying the following equation (eq. (1)) [23, 24]:

(1) $$\text{Water}\;\text{uptake}\;\text{\%}=\frac{\text{Final}\;\text{weight}-\text{Initial}\;\text{weight}}{\text{Initial}\;\text{weight}}\times 100$$

### 2.5 FTIR spectroscopy

The Fourier transform infrared (FTIR) spectrophotometer was used to identify the functional groups of fabricated composites. An attenuated total reflectance (ATR) attached FTIR spectrometer machine (model: IRPrestige 21, SHIMADZU) was employed for functional group analysis. In transmittance mode, FTIR spectrum was counted for the wavenumber range of 400-4000cm^−1^ with 30 scans.

### 2.6 Scanning electron microscopy (SEM)

The fracture surface of the composite, after the tensile test, was explored by scanning electron microscopy (SEM) (model: Phenom Pro). Samples were fixed on a carbon tap and coated with gold for 20 s. All the images were captured at 5 KV voltage.

## 3 Results and discussion

### 3.1 Mechanical properties of the composite

**Figure 1:**
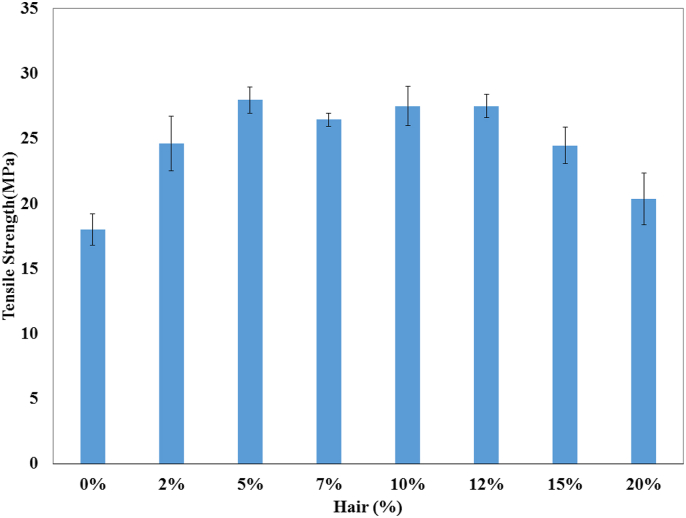
Tensile strength of unsaturated polyester resin and composite with different hair percentages.

**Figure 2:**
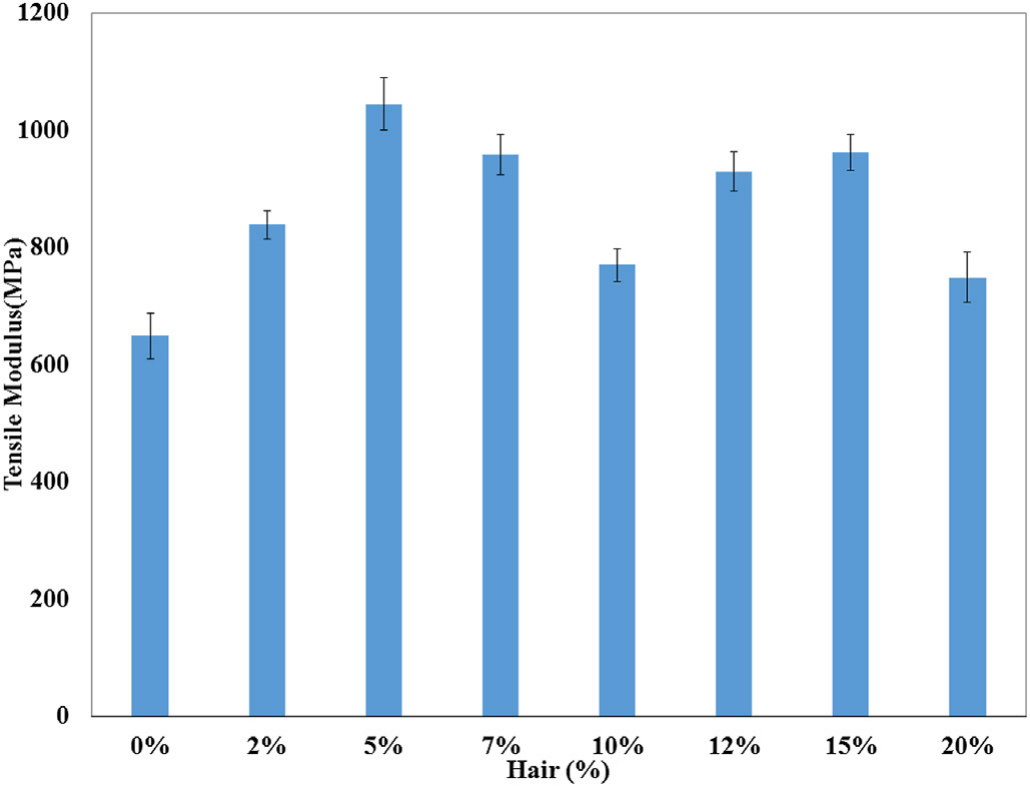
Tensile Modulus of unsaturated polyester resin and composite with different hair percentages.

**Figure 3:**
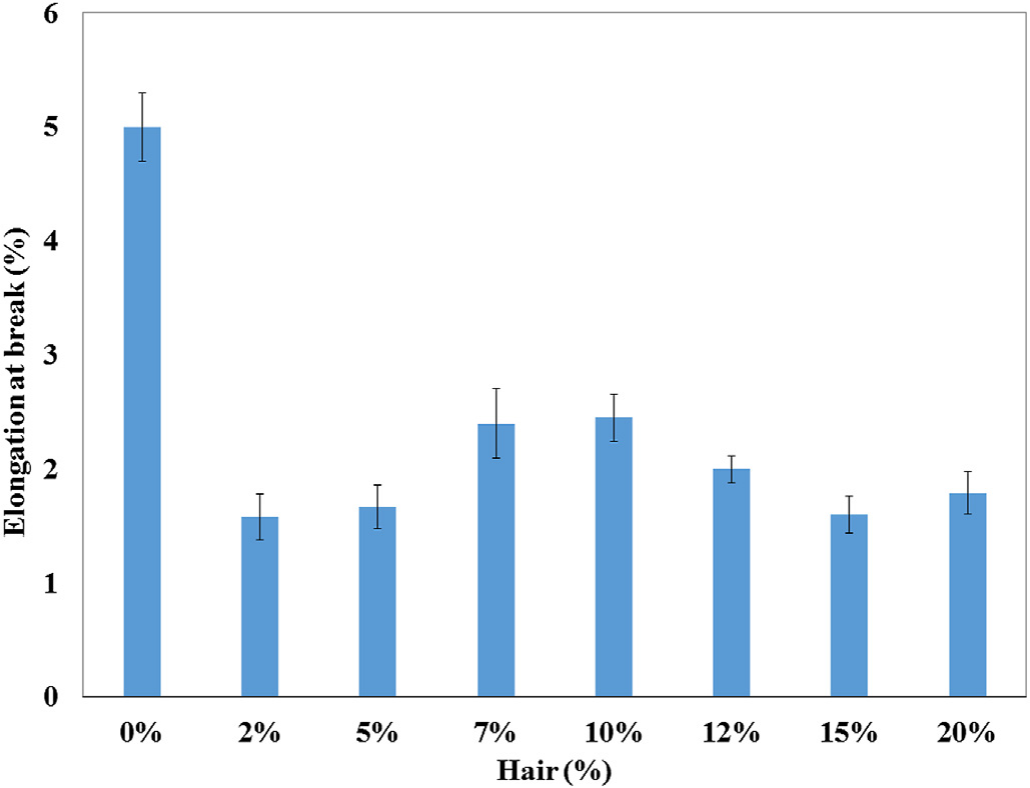
Elongation at break (percentage) of unsaturated polyester resin and composite with different hair percentages.

**Figure 4:**
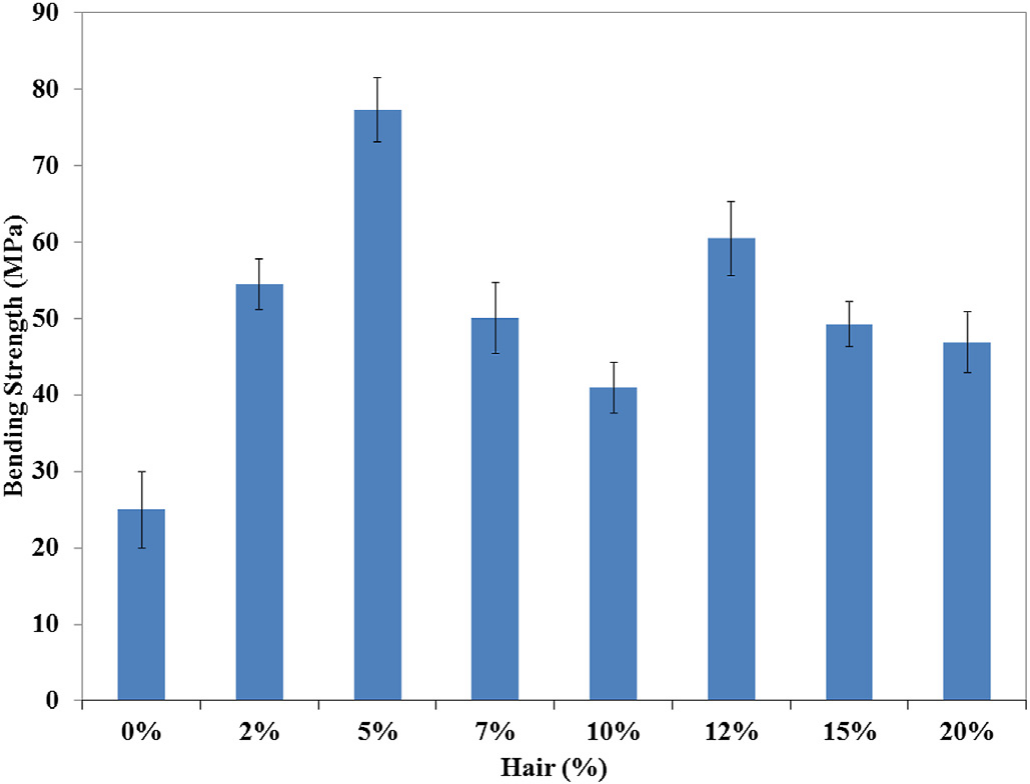
Bending strength of unsaturated polyester resin and composite with different hair percentages.

**Figure 5:**
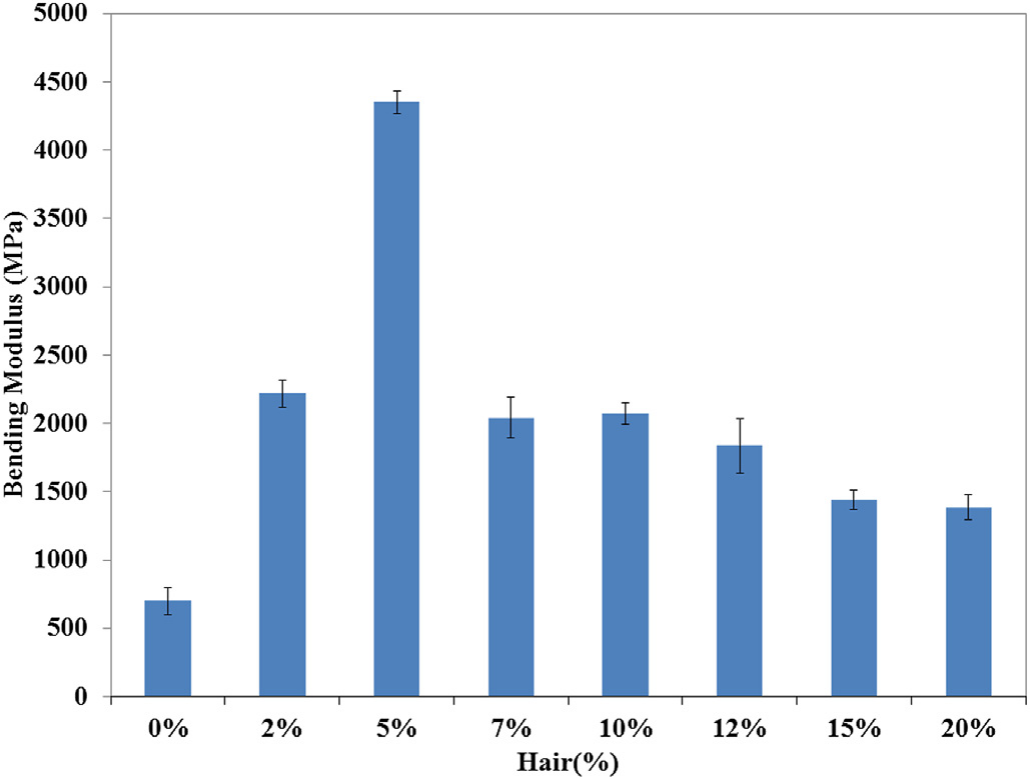
Bending Modulus of unsaturated polyester resin and composite with different hair percentages.

The TS, TM, Eb, BS, and BM of the prepared composites were analyzed by a universal testing machine and the data are illustrated in Figures 1, 2, 3, 4, and 5. These mechanical properties are counted for better applications in various environmental conditions [30, 31]. The values of TS, TM, and EB for the control sample (only UPR) are 18 MPa, 650 MPa, and 5% which are presented in respective figures.

The highest Results of TS and TM were achieved for the 5% cow's hair fiber based-composite and the TS, and TM values were 55.38%, and 60.94% higher than that of the control sample. More addition of cow hair fiber to the composite lessened the stress transfer between the hair and the matrix. On the other hand, low fiber percentage cannot contribute sufficient amount of strength to the composites.

That is why the 2% and 7%–20% hair fiber based-composites revealed lower TS and TM values. Percentage elongation at break for the control sample was higher (5.00%) than that of the fiber loaded-composites. Among the fabricated composites, the 10% fiber based-composites presented the best result, and that value was only 50.98% lower than the control sample.

The incorporation of the cow hair fiber to the unsaturated polyester resin augmented the bending strength. The data are represented in Figure 4 as a column chart. From the figure, it is easily noticeable that the 5% fiber loading composites depicted the best bending strength (77.39 MPa), which was 209.57 % more than that of the control sample. At low fiber percentage (2%), composites cannot gain sufficient strength, while higher percentages (7%, and more) created a problem regarding stress transfer.

The consequence of the cow hair fiber on the BM of prepared composites is illustrated in Figure 5. The 5% cow hair fiber based-composites expressed the best BM values, which was 521.75% higher than that of the control sample. A similar effect, which was previously discussed as in the case of TS and TM, was assumed for BS and BM. This finding also carried good evidence to be a more stable composite than the control sample (UPR). A Similar trend in mechanical properties was reported elsewhere [14, 23, 32, 33, 34, 35].

### 3.2 Water uptake of composites

**Figure 6:**
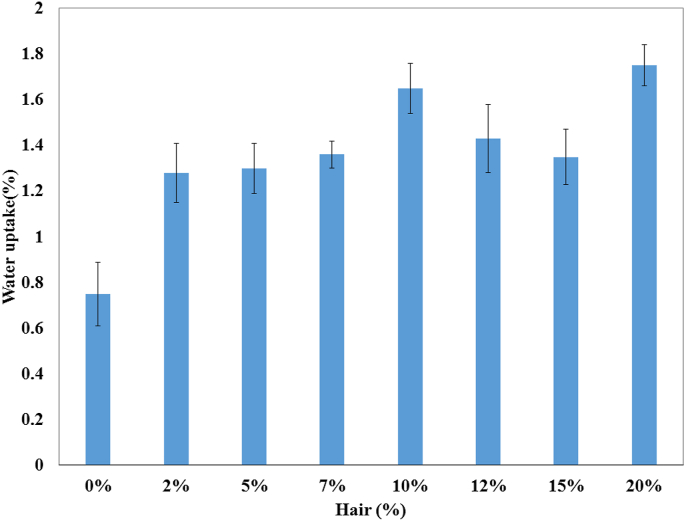
Percentage of water uptake of hair fiber-reinforced composites.

The water uptake influences the composites' water-swelling behavior. Three measurements of each composite were conducted, and their average percentage values were documented. The water uptake findings are displayed in Figure 6 against cow hair fiber percentage at room temperature. The water absorption of the composite varied between 1.28% and 1.75%, which was higher than that of the control sample. The hydrophilic character of natural fiber is a big concern, as it impedes successful outdoors application. The higher content of cow hair fibers in composites offers an extra advantage of good mechanical properties, but the escalation of hydrophilicity leads to further water absorption. The water absorption was increased with time and, no substantial change was documented after 24 h. Similar Results are reported elsewhere [36].

### 3.3 FTIR analysis

**Figure 7:**
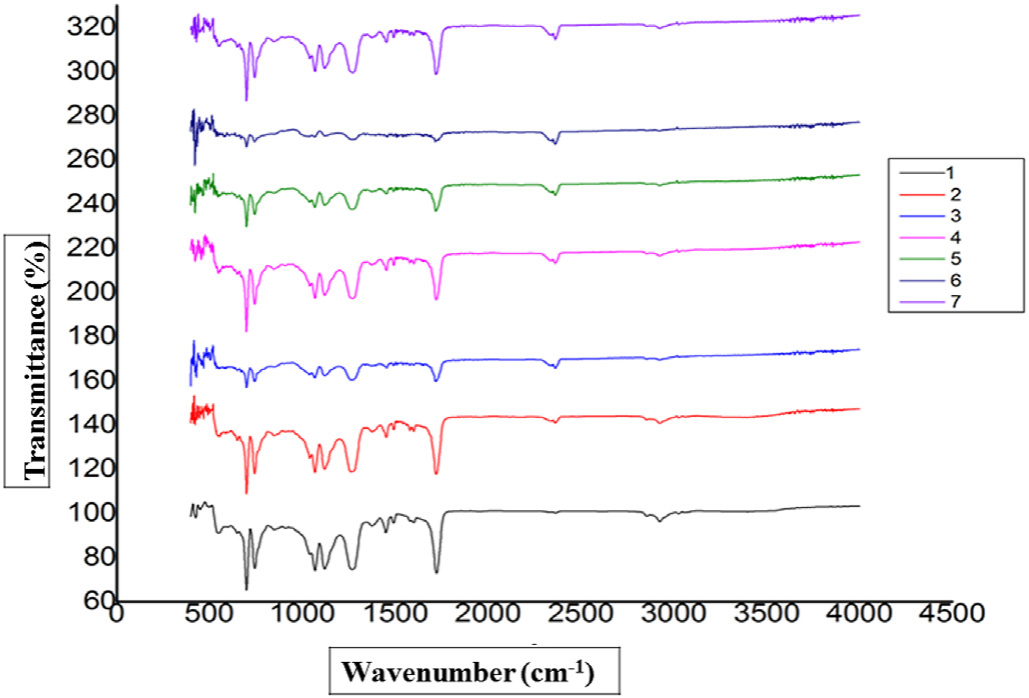
Functional group analysis of prepared composites [illustrated as 1 for 2%, 2 for 5%, 3 for 7%, 4 for 10 %, 5 for 12%, 6 for 15%, and 7 for 20%].

The FTIR analysis was conducted for functional group identification in the Hair-UPR composites. The peak in the wavenumber of 2900 cm^−1^ appeared for the existence of the – NH_2_ group. A further intense peak was observed in the wavenumber of 1700 cm^−1^, which was due to the presence of CH_3_ and CH_2_ groups (for sp3 stretching). Another peak was recognized for CH groups (for sp3 stretching) was witnessed in the area of 2800 cm^−1^. For the sp^3^ bending vibration of CH_3_, CH, and CH_2_ groups, a peak was detected in the wavenumber of 1575 cm^−1^. Another peak for the presence of the C=O group was experimented at 1690-1750 cm^−1^ wavenumber. For the appearance of the OH group, a peak was emerged in the wavenumber of 3400 cm^−1^. All the peak positions were more or less similar to the literature [37, 38, 39, 40]. FTIR data of hair fiber-reinforced UPR based-composites are represented in Figure 7. No extra functional groups were noticed in FTIR spectra, which carried good evidence against the chemical bonding between fiber and UPR. The slightly improved mechanical properties may be the only reason for mechanical bonding between the fiber and matrix.

### 3.4 Scanning electron microscopy analysis

**Figure 8:**
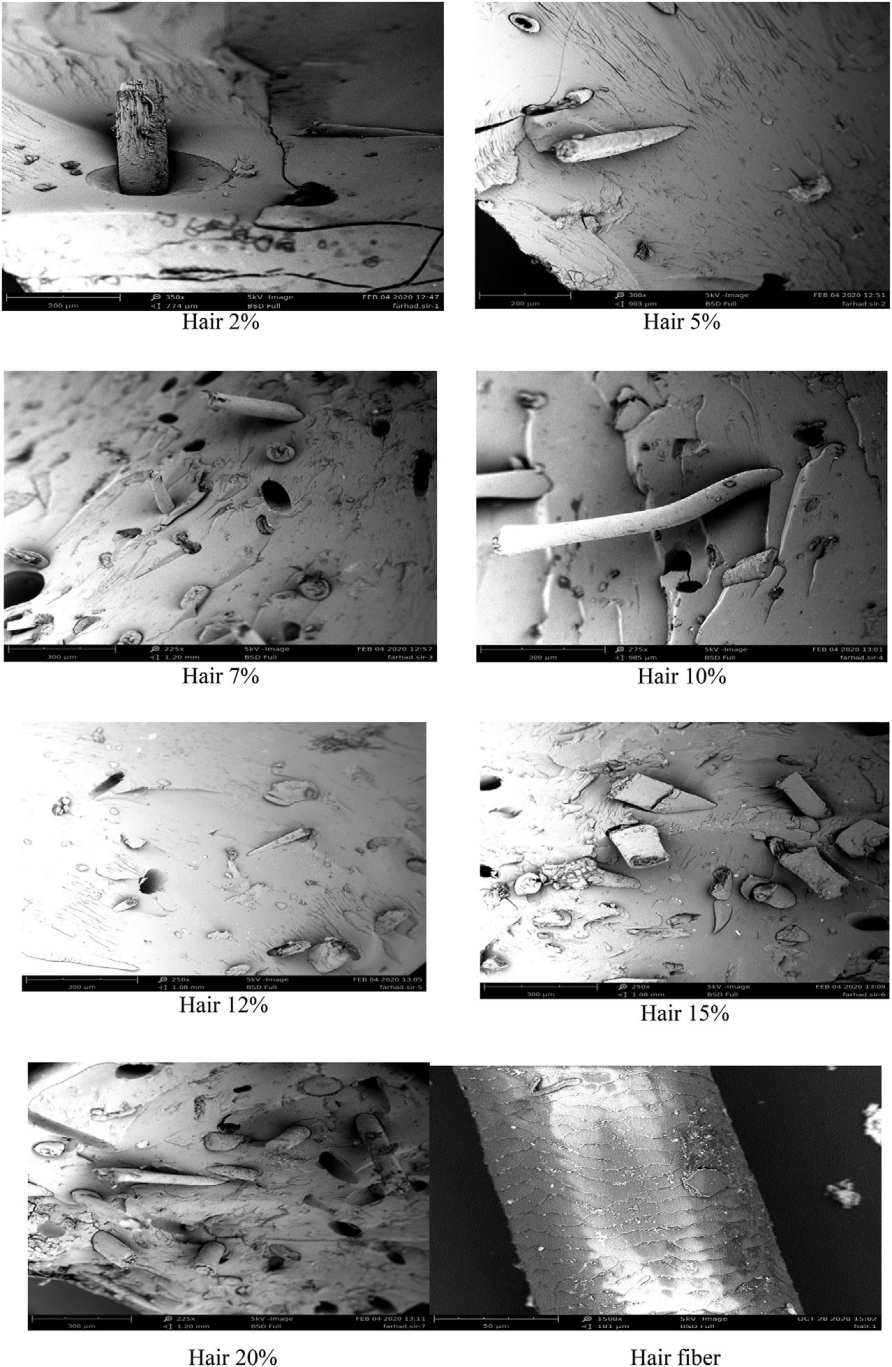
SEM images of different hair based composites and hair fiber.

To analyze the cow fiber and matrix adhesion in the composites, the broken samples (after tensile test) were selected for the analysis of surface morphology under a SEM machine. Figure 8 revealed the SEM images of the fracture surface of the cow hair fiber-reinforced UPR-based composite. For a better understanding of surface morphology, all the samples (2, 5, 7, 10, 12%, 15, and 20% of hair) were analyzed under a SEM machine, and a strong mechanical bonding was noticed between the cow hair fiber and unsaturated polyester resin. The SEM images also disclosed that the pulling of the hair fiber from composites was very poor. A smooth surface was observed for all the composites, and a similar surface was described elsewhere [18, 41]. Some pores, which originated from the fiber pull-our, are noticed in the SEM images. The fiber and matrix are well bound together, which bears clear proof that the mechanical properties of composites are marginally higher than that of the control sample. Similar fiber and matrix adhesion was also experimented within natural fiber-reinforced composites and discussed elsewhere [38, 42, 43]. Generally, there remains no crack in the natural fiber-reinforced composites [35]. As the SEM images were taken after tensile testing, some cracks were observed.

Physico-mechanical properties as well as the environmental issue were the main focus of this research. Up to a certain percentage, the properties were amplified by the addition of fiber. When cellulose based-fibers are gradually added to the matrix (unsaturated polyester resin), the force transfer from the matrix to the reinforcing agent is improved [14, 23]. Our Results also revealed the same trend, and all the findings are discussed in the results and discussion section. The better force transfer between the fiber and matrix amplified the mechanical properties to some extend. There was no chemical bonding between the hair fiber and UPR, which was confirmed from the FT-IR spectrum by exhibiting no new bond. This type of mechanical bonding has been also reported in cellulose-based fiber-reinforced composites [44]. The SEM image of the hair fiber depicted that the surface of the fibers was not entirely smooth, which made the mechanical bonding strong. SEM images of composite materials, where the tensile test pulls a few cow hair fibers from the UPR, indicated the strong bonding between cow hair and UPR. Thus the control utilization of waste materials, cow hair fiber, from leather industries with the unsaturated polyester resin can improve the physico-mechanical properties. Though the mechanical properties and water uptake properties were not change into a vigorous scale, the percentages they amplified the properties were enough for the efficient applications of composites. In addition to this, the utilization of waste materials will reduce environmental pollution.

## 4 Conclusion

Cow hair fiber is available in this country and is a waste material in the leather industry. The use of cow hair fibers is a new, cost-effective, environmentally friendly, and recyclable type of content in the composite process. Cow hair fiber can be used in making environmentally friendly plastic goods for a range of uses and can be quickly replaced with cellulose-based natural fiber. The inclusion of these animal fibers in the matrix enhanced the mechanical properties of the fabricated composite materials. A certain percentage of (5%) cow hair fiber-reinforcement in UPR amplified the properties than that of the control sample or other percentages. For better mechanical properties, it is suggested from this research to use the 5% cow hair fiber-reinforced unsaturated polyester resin based-composites instead of only unsaturated polyester resin.

## Declarations

### Author contribution statement

Md. Farhad Ali: Conceived and designed the experiments; Performed the experiments; Analyzed and interpreted the data; Wrote the paper.

Md. Sahadat Hossain: Analyzed and interpreted the data; Wrote the paper.

Samina Ahmed: Conceived and designed the experiments; Analyzed and interpreted the data; Contributed reagents, materials, analysis tools or data; Wrote the paper.

A. M. Sarwaruddin Chowdhury: Conceived and designed the experiments; Wrote the paper.

### Funding statement

This research did not receive any specific grant from funding agencies in the public, commercial, or not-for-profit sectors.

### Data availability statement

Data included in article/supplementary material/referenced in article.

### Declaration of interests statement

The authors declare no conflict of interest.

### Additional information

No additional information is available for this paper.
